# Experiences of solitude in adulthood and old age: The role of autonomy

**DOI:** 10.1177/01650254221117498

**Published:** 2022-08-24

**Authors:** Jana Nikitin, Fiona Sophia Rupprecht, Christina Ristl

**Affiliations:** University of Vienna, Austria

**Keywords:** Aloneness, self-determined, daily experience, subjective well-being

## Abstract

Recent evidence suggests that older adults experience momentary states of spending time alone (i.e., solitude) less negatively than younger adults. The current research explores the role of autonomy as an explanation mechanism of these age differences. Previous research demonstrated that solitude can be experienced positively when it is characterized by autonomy (i.e., the own wish or decision to be alone). As older adults are relatively more autonomous in their daily lives, they might experience solitude less negatively (in terms of subjective well-being, social integration, self-esteem, and valence) than younger adults. We tested this hypothesis in three studies. In two experience-sampling studies (Study 1: *N* = 129, 59.7% women, age 19–88 years; Study 2: *N* = 115, 66.4% women, age 18–85 years), older age and higher autonomy were associated with more positive experience of everyday solitude moments. Although autonomy did not differ between younger and older adults, perceived (lack of) autonomy partly played a more important role for the experience of solitude moments in younger adults compared to older adults. Finally, Study 3 (*N* = 323, 52% women, age 19–79 years) showed that the relationship between recalled solitude moments of high versus low autonomy and solitude experience is fully explained by feelings of autonomy. Overall, our results demonstrate that older people do not experience more autonomy in situations of solitude than younger adults, but that they partly better cope with low-autonomy solitude. However, people of all ages seem to benefit more from high-autonomy moments of solitude.

Solitude is generally defined as “a state of being alone or remote from society” (Ost Mor et al., 2020, p. 1). Solitude can be challenging because humans are social beings and have a strong need for interpersonal attachments (Baumeister & Leary, 1995). Accordingly, solitude has been frequently associated with negative consequences such as loneliness (Long & Averill, 2003), low positive affect (Pauly et al., 2018), or negative thoughts (Lay et al., 2019). On the contrary, it has been recognized that solitude has also potential benefits such as freedom of choice, relief from social stressors, or opportunity for spirituality or creativity (Long & Averill, 2003). One of the main factors that are associated with the positive experience of solitude is autonomy. When solitude is self-determined, derived by self-motivation, it is experienced positively; non-self-determined solitude that occurs without one’s control and desire is experienced negatively (Ost Mor et al., 2020; Thomas & Azmitia, 2019). In other words, positively experienced solitude is usually a matter of choosing to be by oneself and is experienced as freedom from external constraints (Long & Averill, 2003), freedom from burdens of daily life (Lay et al., 2019), and reduction of stress (Nguyen et al., 2017).

As people get older and their social networks shrink, solitude in daily life increases (Marcum, 2013; Pauly et al., 2016). In addition, older adults have higher preference for solitude than younger adults (Toyoshima & Sato, 2017), suggesting that solitude in older adulthood can be a matter of choice. In fact, the experience of solitude has been linked to more favorable affective and biological outcomes in older adults compared to middle-aged and younger adults (Pauly et al., 2016; Toyoshima & Sato, 2019). These differences have been discussed in terms of better emotion-regulation strategies of older adults (e.g., Hoppmann et al., 2021). Older adults do not only show higher emotional stability and higher motivation to maintain positive affect in daily life (Riediger et al., 2009; Röcke et al., 2009), but also have better emotion-regulation strategies than younger adults (Charles & Carstensen, 2007). Consequently, older people might experience solitude less negatively because they can better deal with negative emotions that might arise from being alone, or because they use solitude for self-reflection and emotion regulation (Hoppmann et al., 2021).

Alternatively, the more positive experience of solitude in older adulthood could reflect differences in life circumstances of younger and older adults (Ost Mor et al., 2020; Pauly et al., 2016; Toyoshima & Sato, 2017). Previous research found that people gain autonomy—which is defined as a sense of self-determination and being free from external pressures in one’s life (Ryff & Keyes, 1995)—as they get older. They become free from external obligations and constraints that are characterized mainly by work and family duties (Bildtgård & Öberg, 2017) and they are less regulated by age-related norms and expectations (Freund et al., 2009). Both provide them with more freedom to pursue the goals they want to. Accordingly, research documented that age is positively associated with autonomy in everyday tasks as well as personal goals (Job et al., 2018; Sheldon et al., 2006).

High autonomy promotes the experience that one has the capacity and motivation to behave consistent with one’s goals, preferences, values, and interests (Deci & Ryan, 2008). In other words, behavior regulation is more harmonious and less prone to conflict. This is also true for daily solitude moments. Higher autonomy in solitude moments promotes behavior that is based on one’s goals, preferences, values, and interests and, consequently, renders the experience of solitude more positive (e.g., Chua & Koestner, 2010). Taking together, older adults’ moments of solitude may be more often self-determined, and thus, more likely to promote the beneficial aspects of being alone (Pauly et al., 2016). In other words, age differences in the experience of solitude might be explained by the degree of autonomy in choosing to be alone.

Based on these theoretical considerations and empirical evidence, we hypothesize that older adults (a) report higher autonomy in moments of solitude and (b) experience moments of solitude more positively than younger adults. In addition, we argue that (c) autonomy is associated with a more positive experience of solitude moments. Finally, we explore whether autonomy is age-differentially related to the experience of solitude moments. In other words, we explore whether it is equally relevant for younger and older adults’ experience that their moments of solitude are self-determined. Although people strive to maximize control across the entire life span, the actual capacity to actively shape their development according to their goals, preferences, values, and interests declines as people age (e.g., due to health constraints; Heckhausen et al., 1989). Developmental theories of self-regulation (e.g., Heckhausen et al., 2010) suggest that people adjust their control strategies accordingly. For example, they downgrade their goals, preferences, values, and interests to the available resources and they use emotion-regulation strategies or downward social comparisons to preserve resources and maintain high levels of emotional well-being and self-esteem. Adopting to solitude, it is possible that autonomy in the choice of solitude moments plays a relatively less relevant role for older adults’ experience because older adults can cope better with situations of limited control. We test this possibility in the present research.

## The Present Research

In line with previous research, we operationalized autonomy as the wish for or one’s own choice of the situation of solitude (Lay et al., 2020; Toyoshima & Sato, 2019).^1^ Solitude was operationalized as having no social interaction in Study 1 and being alone (i.e., without any physical contact to another person) in Study 2 and 3. As outcome variables, we investigated social and (self-)evaluative components of the experience (i.e., state self-esteem, feelings of social integration, and valence of the situation) as well as subjective well-being. These outcomes are not only relevant indicators of affective and interpersonal aspects of people’s daily lives, but might be also affected by autonomy in solitude situations. In fact, previous research has demonstrated that autonomy leads to high levels of state self-esteem (Hodgins et al., 2007), feelings of social integration (i.e., low levels of loneliness; Chua & Koestner, 2010), and positive experience of solitude (Nguyen et al., 2017). These positive effects are generally explained by autonomy promoting focus on one’s goals, preferences, values, and interests, and consequently resulting in a satisfying state of “simply being who one *is*” (Hodgins et al., 2007, p. 189). This should also result in high subjective well-being in solitude situations characterized by high autonomy (Yu et al., 2017). We investigated our hypotheses in two experience-sampling studies assessing daily experience of autonomy and solitude situations in adults of different ages (Study 1 and 2) and in a study (Study 3) assessing experience of recalled solitude situations characterized by high versus low autonomy. Study 1 was approved by the ethics committee of the Faculty of Arts and Social Sciences of the University of Zurich (dated 25 July 2017). Study 2 used the same design as Study 1. Study 3 was conducted according to the ethical principles of the Faculty using a checklist for studies regarding their ethical safety.

## Study 1

Study 1 tested hypotheses (a)–(c) in everyday moments of solitude. In addition, we investigated the age-differential role of autonomy for the experience of everyday moments of solitude. In all three studies, participants provided informed consent for the participation.

### Method

#### Sample

The convenience sample for Study 1 was recruited from Basel and surrounded areas. Out of initially 329 participants, 28 were excluded based on withdrawal from the study or conspicuous fast completion of the lab questionnaire.^2^ Subsequently, 172 individuals (57.1%) were excluded from analyses because they did not report a single experience of solitude. The remaining 129 individuals did not differ significantly from the excluded 172 individuals in terms of age, gender, education, relationship status, household size, self-reported physical, and self-reported psychological health. The final sample of 129 individuals was aged 19–88 years (*M* = 47.82 years, standard deviation (*SD*) = 19.48 years), 59.7% were women, and 23.3% reported university education. Regarding relationship status, 32.6% were married, 24.8% were unmarried but in a long-term relationship, 24.8% were single, 4.7% widowed, 9.3% divorced, and 3.9% in an open relationship. Altogether, 26.4% of the sample reported living alone. Self-reported physical and psychological health were high with means of 4.47 (*SD* = 1.21) and 4.54 (*SD* = 1.30) on a scale ranging from 0 = *very bad* to 6 = *very good*.

#### Daily Assessments

Daily assessments were conducted as paper-and-pencil eight times per day for 3 days using an interval-based experience sampling method (e.g., Bolger & Laurenceau, 2013): Every 90 min, participants received a signal and were instructed to describe their last social interaction. A social interaction was defined as any encounter with at least one person in which the behavior of the participant and the behavior of the other person(s) relate to each other. The mere presence of another person was not included in this definition. If the participants did not experience any social interaction, they were instructed to report their current situation. These entries were used in the current analyses. The 129 participants completed a total of 2,944 entries, *M* = 22.84 (*SD* = 2.29). Demographics were unrelated to the number of entries. Participants reported social interaction in 2,359 (80.1%) entries. The remaining 585 entries (19.9%) were solitude entries. The average person reported 4.54 solitude entries (*SD* = 4.24), with numbers ranging from 1 to 22. The absolute number of solitude entries was positively related to age, *r* = .19, *p* = .035, but unrelated to other demographics. For the current research questions, we analyze the constructs subjective well-being, self-esteem, social integration, and valence of the situation, as well as autonomy. Descriptives and reliabilities of the scales are presented in Table 1.

**Table 1.** Descriptives and Reliabilities of the Outcome Variables.

	Descriptives *M* (*SD*)			Reliabilities (within individuals)		
	Study 1	Study 2	Study 3	Study 1	Study 2	Study 3
Well-being	4.09 (1.23)	2.45 (0.70)	3.70 (1.62)	.95 (.80)	.93 (.80)	.92
Self-esteem	4.16 (1.18)	4.12 (1.22)	3.98 (1.50)	.96 (.72)	–	.87
Social integration	3.68 (1.20)	2.19 (0.77)	3.28 (1.68)	.86 (.84)	–	.88
Valence_Situation/Solitude_	4.37 (1.44)	2.62 (0.92)	3.83 (2.08)	–	–	–

*Notes. M*: mean; *SD*: standard deviation. Reliabilities were determined as within- and between-person alphas using multilevel confirmatory factor analyses. *N_Study 1_* = 129. *N_Study 2_* = 115. *N_Study 3_* = 323. In Study 2, well-being, social integration, and valence were assessed on scales ranging from 0 to 4. All other constructs were assessed on scales ranging from 0 to 6.

##### Outcome Variables

Subjective well-being, state self-esteem, and social integration were asked with the question “How did you feel in the situation?” and assessed with semantic differentials on a 7-point response scale ranging from 3 on the left side to 3 on the right side. For the following analyses, we recoded the response scales to 0–6. The respective items were averaged into one scale score. Lower scale scores presented lower well-being, lower self-esteem, and lower social integration, respectively. Subjective well-being was assessed by the adjectives weary–awakened, balanced–tensed, dissatisfied–satisfied, listless–highly motivated, peaceful–irritated, unhappy–happy (adopted from Schallberger, 2005). Participants reported their current self-esteem using three semantic differentials developed by the authors (insecure–self-assured, incompetent–competent, self-confident–abashed). Two semantic differentials assessed social integration versus social isolation (rejected/isolated–loved/liked and lonely–fully integrated; adopted from Nikitin & Freund, 2018). Valence of the situation (Schallberger, 2005) was assessed by one item (negative–positive) with the same anchors as the other variables.

##### Autonomy

The emergence of the situation was asked with the question “What led to the situation and to what extent?” Participants reported on a Likert-type scale ranging from 0 = *not at all* to 6 = *very much* whether the situation emerged due to “my own wish/decision” (*M* = 4.22, *SD* = 2.05).

#### Analyses

In a first step, we investigated whether older adults experienced higher autonomy in situations of solitude than younger adults. In a next step, we investigated how younger and older individuals experienced situations of solitude regarding the four outcome variables through linear multilevel modeling. On Level 2, we modeled individual averages of the outcome variables across situations and added age, the individual average autonomy, and the individual percentage of solitude entries reported (e.g., 8% out of overall 24 entries) as predictors. On Level 1, we modeled situational deviations from those averages in well-being, self-esteem, social integration, and valence and entered situational autonomy as a predictor to the model. We allowed for a random slope of situational autonomy and added an interaction term between age and situational autonomy as an additional predictor. The amount of variance explained by the predictors in the random intercept, residual, and the random slope of situational autonomy are given together with the remaining variance unexplained by the predictors. Hereby, predictors on Level 2 explain variance in the random intercept, predictors on Level 1 (as well as the presence of the random slope of situational autonomy) can explain variance in the residual, and the cross-level interaction of age and situational autonomy can explain variance of the random slope of situational autonomy.

As the inclusion of gender and university education (relevant age-related predictors of well-being; e.g., Pinquart & Sörensen, 2001) did not change the results, analyses are reported without these covariates. Analyses were conducted with R 4.1.1 (R Core Team, 2021) and the packages reghelper and lmerTest (Hughes, 2021; Kuznetsova et al., 2017).

### Results and Discussion

**Figure 1.**
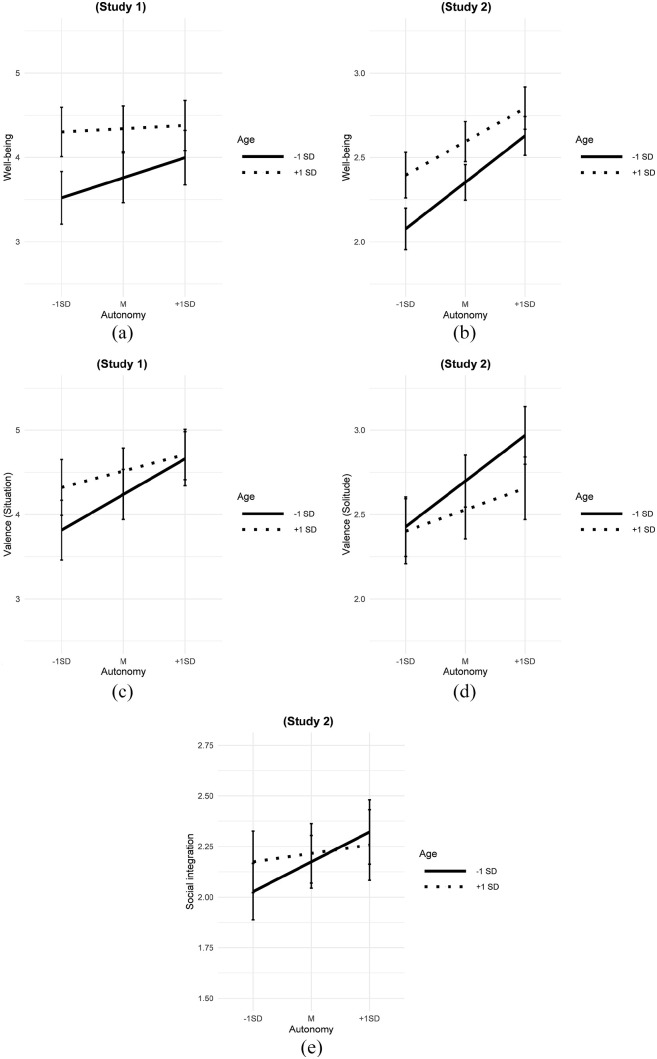
Interactions Between Age and Situational Autonomy from Studies 1 and 2. *Note.* Error bars indicate 95% confidence intervals. *N_Study 1_* = 129. *N_Study 2_* = 115.

Correlations of the study variables within and between individuals can be found in Supplementary Table S1. Intraclass correlation coefficients ranged from .43 to .59 for the outcome variables and the autonomy construct, justifying our multilevel modeling approach. A first multilevel regression indicated that older adults did not experience more autonomy in situations of solitude than younger adults, γ = –0.00, standard error (*SE*) = 0.01, *p* = .629.^3^ Table 2 shows the main analyses. Older adults expressed higher well-being, self-esteem, and social integration in situations of solitude than younger adults. Higher average feelings of autonomy were associated with higher well-being, higher social integration (marginally), more positive valence, but not higher self-esteem in general (explained variance in the random intercept: 13.3%, 7.8%, 21.5%, and 9.8%). Situational autonomy was related to higher situational well-being, self-esteem, and more positive valence (explained variance in the residual: 16.2%, 17.1%, and 37.9%). Age interacted significantly with situational autonomy in predicting its association with well-being and valence (explained variance in the random slope of situational autonomy: 54.2% and 27.9%). As can be seen in Figures 1(a) and (c), older adults’ reports of well-being and valence were less strongly associated with low-autonomy situations of solitude.

**Table 2.** Multilevel Regression Analyses on the Experience of Daily Situations of Solitude—Study 1.

Outcome variable	Well-being	Self-esteem	Social integration	Valence _Situation_
Fixed effects	Standardized coefficient [95% CI]			
Individual level (Level 2)				
Age	**0.23** [0.08, 0.38]	**0.22** [0.07, 0.38]	**0.21** [0.06, 0.37]	0.10 [−0.04, 0.23]
Proportion of solitude	0.14 [−0.03, 0.30]	0.16 [−0.01, 0.34]	**0.24** [0.06, 0.41]	0.13 [−0.01, 0.28]
Autonomy_Average_	**0.26** [0.12, 0.40]	0.12 [−0.03, 0.26]	***0.14*** [−0.01, 0.28]	**0.35** [0.23, 0.48]
Situational level (Level 1)				
Autonomy_Situation_	**0.11** [0.04, 0.18]	**0.07** [0.02, 0.13]	0.05 [−0.03, 0.13]	**0.21** [0.13, 0.30]
Cross-level Interaction				
Age × autonomy_Situation_	**−0.08** [−0.15, –0.02]	−0.01 [−0.07, 0.04]	−0.06 [−0.13, 0.02]	**−0.08** [−0.15, –0.00]
Random effects	Variance (ΔR^2^)			
Intercept	0.767 (13.3%)	0.794 (9.8%)	0.811 (7.8%)	0.722 (21.5%)
Residual	0.584 (16.2%)	0.519 (17.1%)	0.449 (28.3%)	0.884 (37.9%)
Slope_Autonomy Situation_	0.011 (54.2%)	0.001 (–)	0.024 (11.1%)	0.030 (27.9%)

*Notes.* CI: confidence interval. Significant parameters (*p* < .05) are printed in bold. Parameters approaching significance (*p* < .07) are printed in cursive. *N_Study 1_* = 129. Unstandardized coefficients are reported in the supplemental materials. For the random effects, remaining variances unexplained by the predictors are given outside the parentheses. The amount of variance explained by the predictors is given inside parentheses.

To summarize, Study 1 partly supported the hypotheses. Moments of solitude were experienced more positively by older compared to younger adults in terms of higher subjective well-being, self-esteem, and social integration. Unexpectedly, autonomy was not associated with age, ruling out the possibility that higher levels of autonomy can account for the age differences in solitude experience. Autonomy was, however, associated with the experience of solitude both on the individual and the situational level. Finally, older adults’ experience of the situation in terms of subjective well-being and valence (but not self-esteem and social integration) was less strongly connected to autonomy, suggesting that autonomy might play a partly weaker role in older adulthood. To validate these findings, we conducted a second experience-sampling study.

## Study 2

Study 2 aimed to validate the results of Study 1 in a more extensive research design covering up to 42 (instead of Study 1’s up to 24) situations across a longer period of time (7 instead of 3 days). In addition, participants in Study 2 were instructed to report their current situation, capturing a higher number of naturally occurring solitude situations than in Study 1, which focused on social interactions. Solitude was also defined more strictly than in Study 1 as a situation in which no other person was present. Finally, valence was operationalized specifically as valence of the solitude (rather than the situation in general). Again, we tested hypotheses (a)–(c) and explored the age-differential role of autonomy for the experience of everyday moments of solitude in regard to subjective well-being, self-esteem, social integration, and valence.

### Method

#### Sample

The convenience sample for Study 2 was recruited from Vienna and surrounded areas. Out of 117 initial participants, 2 individuals were excluded from analyses because they did not report a single experience of solitude. The final sample of 115 individuals was aged 18–85 years (*M* = 37.90 years, *SD* = 16.56 years), 66.4% were women, and 58.4% reported university education. Regarding relationship status, 25.7% were married, 37.2% were unmarried but in a long-term relationship, 28.3% were single, 2.7% widowed, 3.5% divorced, and 2.7% in an open relationship. Altogether, 23.9% of the sample reported living alone. Self-reported physical and psychological health were high with means of 4.22 (*SD* = 1.34) and 4.07 (*SD* = 1.41) on a scale ranging from 0 = *very bad* to 6 = *very good*.

#### Daily Assessments

Daily assessments were conducted via a self-programmed app six times per day for 7 days. Participants received a randomized signal in 2-hr intervals (between 8 am and 8 pm) and were instructed to describe the current situation. The 115 participants completed a total of 2,966 entries, *M* = 25.79 (*SD* = 12.04). Older adults completed more entries than younger adults, *r* = .32, *p* < .001. The other demographics were unrelated to the number of entries. Participants reported the presence of another person in 1,864 (62.8%) entries. The remaining 1,181 entries (37.2%) were solitude entries and were used for analyses. The average person reported 10.27 solitude entries (*SD* = 6.83), with numbers ranging from 1 to 30. The absolute number of solitude entries was positively related to age, *r* = .22, *p* = .016, and was linked to the relationship status of the individual, *Chi*^2^ (120) = 151.64, *p* = .027. For the current research questions, we analyze the constructs subjective well-being, self-esteem, social integration, and valence of solitude, as well as autonomy. Descriptives and reliabilities of the scales are presented in Table 1.

##### Outcome Variables

Subjective well-being and social integration were asked with the question “How did you feel in the situation?” and assessed with semantic differentials on 5-point response scale ranging in values from 0–4. Subjective well-being was assessed by the adjectives weary–awakened, balanced–tensed, dissatisfied–satisfied, peaceful–irritated (see Study 1), as well as exhausted–energetic, and unwell–well. Social integration was assessed with the semantic differential lonely–fully integrated (see Study 1). State self-esteem was assessed with the question “How high was your self-esteem in the current situation?” on a scale ranging from 0 = *extremely low* to 6 = *extremely high* (Brailovskaia & Margraf, 2020). To assess valence, participants were asked to rate their experience of solitude on a scale ranging from 0 = *very negative* to 4 = *very positive*.

##### Autonomy

Autonomy was assessed as in Study 1. Participants indicated that their “own wish/decision” lead to the respective situation with a mean of 2.95 (*SD* = 1.04) on a scale ranging from 0 = *not at all* to 6 = *very much*.

#### Analyses

As a first step, we again investigated whether older adults experienced higher autonomy in situations of solitude than younger adults. In the next step, we applied the same multilevel models as of Study 1. As the inclusion of gender and university education did not change the results, analyses are reported without these covariates.

### Results and Discussion

Correlations of the study variables within and between individuals can be found in Supplementary Table S2. Intraclass correlation coefficients ranged from .15 to .52 for the five dependent variables and the autonomy construct, justifying our multilevel modeling approach. A first multilevel regression indicated that older adults did not experience more autonomy in situations of solitude than younger adults, γ = 0.00, *SE* = 0.00, *p* = .510. Table 3 shows the main analyses. Older adults expressed higher well-being and higher self-esteem in situations of solitude than younger adults. Higher average feelings of autonomy were related to higher well-being, higher self-esteem, and more positive valence of solitude in general (explained variance in the random intercept: 24.0%, 13.8%, 22.1%). Situational autonomy was associated with higher situational well-being, self-esteem, social integration, and more positive valence of the solitude experience (explained variance in the residual: 20.0%, 13.2%, 7.3%, 12.5%). Age interacted significantly with situational autonomy in predicting its association with valence and marginally in predicting its association with well-being and social integration (explained variance in the random slope of situational autonomy: 19.7%, 8.5%, 7.5%). As can be seen in Figures 1(b), (d), and (e) and in accordance to Study 1, older adults’ experience of the solitude situation was less strongly connected to autonomy. Thus, despite a slightly different study design, Study 2 largely replicated the results of Study 1, supporting the robustness of the findings.

**Table 3.** Multilevel Regression Analyses on the Experience of Daily Situations of Solitude—Study 2.

Outcome variable	Well-being	Self-esteem	Social integration	Valence_Solitude_
Fixed effects	Standardized coefficient [95% CI]			
Individual level (Level 2)				
Age	**0.18** [0.07, 0.29]	**0.24** [0.10, 0.38]	0.03 [−0.10, 0.15]	−0.10 [−0.22, 0.02]
Proportion of solitude	−0.07 [−0.18, 0.04]	−0.10 [−0.23, 0.03]	−0.06 [−0.17, 0.06]	−0.11 [−0.23, 0.01]
Autonomy_Average_	**0.23** [0.13, 0.33]	**0.16** [0.04, 0.28]	0.04 [−0.07, 0.15]	**0.27** [0.16, 0.38]
Situational level (Level 1)				
Autonomy_Situation_	**0.34** [0.28, 0.39]	**0.20** [0.14, 0.25]	**0.12** [0.06, 0.19]	**0.22** [0.16, 0.28]
Cross-level interaction				
Age × autonomy_Situation_	***–0.05*** [−0.11, 0.00]	−0.04 [−0.09, 0.01]	***–0.07*** [−0.13, 0.00]	**−0.08** [−0.14, –0.02]
Random effects	Variance (ΔR^2^)			
Intercept	0.167 (24.0%)	0.648 (13.8%)	0.195 (–)	0.268 (22.1%)
Residual	0.247 (20.0%)	0.599 (13.2%)	0.366 (7.3%)	0.599 (12.5%)
Slope_Autonomy Situation_	0.011 (7.5%)	0.046 (5.8%)	0.030 (8.5%)	0.033 (19.7%)

*Notes.* CI: Confidence interval. Significant parameters (*p* < .05) are printed in bold. Parameters approaching significance (*p* < .07) are printed in cursive. *N_Study 2_* = 115. Unstandardized coefficients are reported in the supplemental materials. For the random effects, remaining variances unexplained by the predictors are given outside the parentheses. The amount of variance explained by the predictors is given inside parentheses.

## Study 3

Study 3 was conducted to test whether feelings of autonomy can explain differences between recalled high and low-autonomy situations of solitude. We again explored moderating effects of age for the experience of high versus low-autonomy solitude.

### Method

#### Sample and Procedure

Study 3 was conducted online through an agency. Out of the initial 344 participants, 21 were excluded based on incorrect responses to a control item or a self-declared lack of carefulness and accuracy in answering the questions. The final sample comprised 323 individuals aged 19–79 years (*M* = 49.4 years, *SD* = 14.9 years), 52% were women, and 24% reported university education.

After providing informed consent and stating basic demographics, participants were randomly assigned to one of two conditions. In the *high-autonomy* condition (168 participants, 52%), participants were asked to “Think about a situation in which you were voluntarily alone, because you decided or wished for it and not because it just so happened.” In the *low-autonomy* condition (155 participants, 48%), participants were asked to “Think about a situation in which you were involuntarily alone, because it just so happened and not because you decided or wished for it.” Participants were then given time to freely describe their situation based on their behaviors, thoughts, and feelings. Finally, they were then asked to use the same items as in Study 1 to indicate their subjective well-being, self-esteem, social integration, and valence in the situation.

Participants within the two conditions did not differ from each other in regard to age and gender. Participants in the *high-autonomy* condition were, however, more likely to report a university degree, *Chi^2^*(1) = 6.10, *p* = .014.

#### Measures

Situational subjective well-being, self-esteem, social integration, valence, and autonomy were assessed in the same way as of Study 1. Descriptives and reliabilities are depicted in Table 1.

#### Analyses

We calculated multiple regression analyses with well-being, self-esteem, social integration, and valence serving as outcome variables. We investigated the effects of condition and age, as well as the interaction effect of condition × age. We also tested whether self-reported autonomy (see Study 1 and 2) was able to explain the effects of condition. The inclusion of gender and university education did not change the results. Analyses are thus reported without these two covariates.

### Results and Discussion

Individuals in the *low-autonomy* condition reported significantly less autonomy than individuals in the *high-autonomy* condition, *M* (*SD*)_*1*_ = 2.70 (2.37), *M* (*SD*)_*2*_ = 5.10 (1.46), *t*(263.3) = 11.02, *p* < .001, *d* = 1.22. Table 4 depicts the regression analyses. The two conditions significantly differed in situational well-being, self-esteem, and social integration. Furthermore, older adults reported higher well-being, self-esteem, and social integration. There were no significant age × condition interactions, indicating that the differences in experience between high- and low-autonomy condition were independent of age. Including self-reported autonomy as a predicting variable rendered the differences between the conditions in well-being, self-esteem, and social integration insignificant, and substantially reduced the difference in valence. Furthermore, age no longer predicted well-being and self-esteem, when self-reported autonomy was included as a predictor (ΔR^2^ of 28.0%, 20.2%, 25.1%, and 28.9%).

**Table 4.** Regression Analyses on the Remembered Experience of Solitude—Study 3.

Outcome variable	Well-being		Self-esteem		Social integration		Valence _Situation_	
Constructs	Standardized coefficient [95% CI]							
Condition	**0.40** [0.30, 0.50]	0.07 [−0.03, 0.16]	**0.33** [0.23, 0.43]	0.04 [−0.06, 0.15]	**0.35** [0.25, 0.46]	0.03 [−0.07, 0.13]	**0.44** [0.34, 0.54]	**0.10** [0.01, 0.19]
Age	**0.15** [0.05, 0.25]	0.09 [0.01, 0.17]	**0.15** [0.05, 0.26]	0.11 [0.01, 0.20]	**0.21** [0.11, 0.31]	**0.16** [0.07, 0.24]	0.09 [−0.01, 0.19]	0.04 [−0.04, 0.12]
Age × condition	−0.05 [−0.15, 0.05]	−0.02 [−0.10, 0.06]	−0.04 [−0.15, 0.06]	−0.02 [−0.11, 0.07]	−0.04 [−0.14, 0.06]	−0.01 [−0.10, 0.07]	0.00 [−0.09, 0.10]	0.03 [−0.05, 0.11]
Autonomy		**0.62** [0.53, 0.72]		**0.53** [0.43, 0.64]		**0.60** [0.50, 0.70]		**0.63** [0.54, 0.73]
R^2^	16.1%	44.1%	11.2%	31.4%	15.3%	40.4%	18.3%	47.2%

*Notes.* CI: Confidence interval. Significant parameters (*p* < .05) are printed in bold. The intercept pertains to individuals in the low-autonomy condition. *N_Study 3_* = 323. Unstandardized coefficients are reported in the supplemental materials.

Study 3 supported the findings of Study 1 and 2 by demonstrating that solitude that emerges without autonomy is associated with less positive experience compared to solitude which emerges autonomously, because one decided or wished so. Self-reported autonomy fully (or partly in case of valence) mediated the differences between high and low-autonomy conditions, indicating that autonomy is the driving mechanism in these differences. Although age was positively associated with the situational experience, age—in contrast to Study 1 and 2—did not moderate the effects of the manipulation. Instead, age no longer predicted subjective well-being and self-esteem when self-reported autonomy was included as a predictor, suggesting that autonomy was a more important predictor of the differences in solitude experience between low- and high-autonomy solitude than age.

## General Discussion

Over the life span, people experience solitude for many different reasons. In the current research, we investigated the role of autonomy as a predictor of the experience of solitude. We argued that older adults compared to younger adults experience moments of solitude more positively because their everyday lives are more under their own control and their moments of solitude are more often self-determined. Although our studies demonstrated that both age and autonomy are associated with more positive experience of solitude, they did not support our hypothesis that autonomy is the explaining mechanism of age differences in solitude experience. In other words, older adults’ moments of solitude were not more often self-determined than solitude moments of young adults. These results indicate how much people rely on other people in their daily lives and are in line with previous findings showing the fundamental importance of social interactions for people’s lives (e.g., Shor & Roelfs, 2015). As Coplan and colleagues put it, “despite the widely held beliefs that solitude serves self-enhancing functions, it is often experienced as unwelcome and painful” (Coplan et al., 2021, p. 3), which might cause people of all ages to prefer being with others over being alone.

However, solitude that results from situational (as well as average) own wish or decision is associated with a more positive experience. This finding was replicated across three different studies in the present research and is in line with previous research and reasoning (Chua & Koestner, 2010; Hodgins et al., 2007; Nguyen et al., 2017; Ost Mor et al., 2020). Future research should directly test the suggested explanation that high autonomy leads to more positive experience of solitude because people in high-autonomy solitude tend to behave more strongly according to their goals, interests, values, and preferences.

Interestingly, our research also demonstrates that this generally positive association between autonomy and experience of solitude is partly diminished in older adulthood, particularly for subjective well-being and valence of the situation (solitude). This is a finding that indirectly supports developmental theories of self-regulation (e.g., Heckhausen et al., 2010). Specifically, the diminished role of autonomy for the positive experience of solitude in older adulthood might be explained by compensatory mechanisms (e.g., downgrading own aspirations or stronger use of emotion-regulation strategies) in older adults that might result from older adults’ limited capacity to exert control (e.g., based on age-related deterioration in health). In fact, older people who have difficulties with everyday activities might even prefer solitude to *gain* autonomy and control (Hoppmann et al., 2021). Note, however, that the moderating effects of age were not found for all outcome variables in the present studies and they need to be replicated before drawing firm conclusions. In addition, feelings of autonomy were equally important for younger and older adults’ experience of solitude when they recalled situations of high versus low autonomy in Study 3. This might be explained by rather extreme situations that have been recalled in this study (compared to the probably rather mundane everyday situations of solitude that were reported in the experience sampling studies). Distinct situations of high or low autonomy might override age-related differences in emotional regulation and other regulatory strategies associated with older adulthood (for similar results see Job et al., 2018).

With regard to the different outcome variables in the present research, we found relatively reliable evidence for the association between autonomy, age, and age × autonomy interactions for subjective well-being, self-esteem, and valence of the situation and somehow less reliable evidence for social integration. This could indicate that the experience of social integration in moments of solitude is more complex and might be affected by additional factors. In fact, previous research has demonstrated that solitude is experienced less negatively for individuals with higher quality social relationships (Pauly et al., 2018) and it has been argued that positive experience of solitude is only possible when people are securely attached (Littman-Ovadia, 2019). It is an interesting question for future research to further explore for whom and under which circumstances moments of solitude are associated with feelings of high social integration.

Finally, all studies of the present research were correlational in nature and, thus, do not rule out different causality or confounding effects on the results. As previous research demonstrated, there are numerous traits and other variables (such as quality of one’s relationships) that moderate the experience of solitude (Birditt et al., 2019; Lay et al., 2019; Yuan & Grühn, 2020) and might underlie both autonomy and experience of solitude. Although Study 3 supported the explanatory power of self-reported autonomy for the differences in experience of solitude moments of high versus low autonomy, future research is needed to test causality of the predictions.

To conclude, solitude is a complex and paradoxical phenomenon, enabling both positive and negative experience and providing both benefits and pains. The present research suggests that it is important to consider for which reasons solitude emerges in order to understand the different consequences of solitude. It also contributes to our understanding of age-related differences in the experience of solitude by showing that older adults can better cope with low-autonomy solitude than younger adults do.

## Supplemental Material

sj-docx-1-jbd-10.1177_01650254221117498 – Supplemental material for Experiences of solitude in adulthood and old age: The role of autonomy Click here for additional data file. Supplemental material, sj-docx-1-jbd-10.1177_01650254221117498 for Experiences of solitude in adulthood and old age: The role of autonomy by Jana Nikitin, Fiona Sophia Rupprecht and Christina Ristl in International Journal of Behavioral Development

## Notes

1. In addition, we also explored the emergence of solitude due to external constraints as an alternative operationalization of (lack of) autonomy, because the desire or preference for solitude can origin not only from autonomous motivation for solitude but also for other, external reasons. However, in the present studies, external constraints were not as consistent a predictor of solitude experience as the own desire, suggesting that own desire is the more reliable positive predictor for the experience of solitude. We nevertheless report analyses with external constraints in supplemental materials.

2. Participants underwent an intervention that trained them to focus on either the well-being of others or the well-being of themselves (for a detailled description of the procedure see Nikitin & Freund, 2020). The intervention had no influence on the present main findings. Note that the present publication used the same study as the Nikitin and Freund (2020) publication. However, while the present publication used only data assessing solitude moments, the Nikitin and Freund (2020) publication used only data assessing social-interaction situations.

3. Older adults did, however, experience fewer external constraints in situations of solitude than younger adults. Results pertaining to the constraints construct are reported in the supplement.
