# Supplementary material for: Experiences of solitude in adulthood and old age: The role of autonomy
Source: Int J Behav Dev. 2022 Aug 24;46(6):510–9. doi: 10.1177/01650254221117498 (PMC9650722; doi:10.1177/01650254221117498)
Supplement: sj-docx-1-jbd-10.1177_01650254221117498 – Supplemental material for Experiences of solitude in adulthood and old age: The role of autonomy [file sj-docx-1-jbd-10.1177_01650254221117498.docx]

| **Supplementary Table S1** | | | | | | | |
| --- | --- | --- | --- | --- | --- | --- | --- |
| *Bivariate Correlations for Study 1 Variables* | | | | | | | |
| Variable | 2 | 3 | 4 | 5 | 6 | 7 | 8 |
| 1. Age | .17 | .20 | **.27** | **.28** | .08 | -.10 | **-.36** |
| 2. Proportion of Solitude | – | **.33** | **.25** | **.36** | **.29** | .21 | **-.24** |
| 3. Well-being | – | – | **.81** | **.67** | **.82** | **.34** | **-.39** |
| 4. Self-esteem | – | **.62** | – | **.81** | **.60** | .23 | **-.34** |
| 5. Social integration | – | **.52** | **.37** | – | **.61** | .13 | -.21 |
| 6. Valence | – | **.58** | **.37** | **.39** | – | **.44** | -.15 |
| 7. Autonomy | – | **.24** | .11 | **.19** | **.40** | – | .12 |
| 8. Constraints | – | **-.15** | .06 | **-.20** | **-.23** | **-.26** | – |
| *Note.* Between-individual correlations are depicted above the diagonal, within-individual correlations are depicted below the diagonal. Age and proportion of solitude as between-individual variables were only included in between-individual correlations. Significant correlations (*p* < .05) are printed in bold. *N_Study 1_* = 129. | | | | | | | |

| **Supplementary Table S2** | | | | | | | |
| --- | --- | --- | --- | --- | --- | --- | --- |
| *Bivariate Correlations for Study 2 Variables* | | | | | | | |
| Variable | 2 | 3 | 4 | 5 | 6 | 7 | 8 |
| 1. Age | -.03 | **.31** | **.34** | .06 | .17 | .07 | .02 |
| 2. Proportion of Solitude | – | -.19 | -.19 | -.12 | -.09 | **-.27** | -.09 |
| 3. Well-being | – | – | **.69** | **.71** | **.76** | **.45** | .07 |
| 4. Self-esteem | – | **.56** | – | **.53** | **.44** | **.26** | .22 |
| 5. Social integration | – | **.51** | **.31** | – | **.42** | .19 | .22 |
| 6. Valence | – | **.63** | **.44** | **.26** | – | **.70** | -.05 |
| 7. Autonomy | – | **.41** | **.27** | **.10** | **.49** | – | -.26 |
| 8. Constraints | – | **-.19** | **-.12** | **-.08** | **-.30** | **-.29** | – |
| *Note.* Between-individual correlations are depicted above the diagonal, within-individual correlations are depicted below the diagonal. Age and proportion of solitude as between-individual variables were only included in between-individual correlations. Significant correlations (*p* < .05) are printed in bold. *N_Study 2_* = 115. | | | | | | | |

| **Supplementary Table S3** | | | | | | | | |
| --- | --- | --- | --- | --- | --- | --- | --- | --- |
| *Bivariate Correlations for Study 3 Variables* | | | | | | | | |
| Variable | 1 | 2 | 3 | 4 | 5 | 6 | 7 | 8 |
| 1. Age | – |  |  |  |  |  |  |  |
| 2. Condition | **-.12**** | – |  |  |  |  |  |  |
| 3. Well-being | .09 | **.38**** | – |  |  |  |  |  |
| 4. Self-esteem | **.11** | **.31*** | **.85** | – |  |  |  |  |
| 5. Social integration | **.17** | **.33*** | **.81** | **.78** | – |  |  |  |
| 6. Valence | .04 | **.43**** | **.88** | **.81** | .79 | – |  |  |
| 7. Autonomy | .02 | **.52**** | **.66** | **.56** | .62 | **.69** | – |  |
| 8. Constraint | -.04 | **-.26** | **-.45** | **-.42** | -.44 | **-.43** | **-.44** | – |
| *Note.* Condition was coded as 0 for low constraints and as 1 for high constraints. Significant correlations (*p* < .05) are printed in bold. *N_Study 3_* = 323. | | | | | | | | |

**Supplementary Material S4 – Analyses Including Constraints for Study 1**

Constraints were assessed by asking “What led to the situation and to what extent?” and offering the answering option “constraints of any kind” on a scale ranging from 0 (= *not at all*) to 6 (= *very much*). The mean was 1.96, the standard deviation was 2.18, bivariate correlations are depicted in Supplementary Table S1. A multilevel regression indicated that older adults did experience less constraints in situations of solitude than younger adults, *γ* = -0.02, *SE* = 0.01, *p* = .035.

| **Supplementary Table S4** | | | | |
| --- | --- | --- | --- | --- |
| *Multilevel Regression Analyses Including the Constraints Construct – Study 1.* | | | | |
| Outcome Variable | Well-being | Self-esteem | Social  Integration | Valence _Situation_ |
| Fixed Effects | Coefficient [95% CI] | | | |
| Intercept | **4.11** [4.03, 4.54] | **4.19** [3.93, 4.45] | **3.86** [3.58, 4.13] | **4.37** [4.10, 4.63] |
|  |  |  |  |  |
| Individual Level (Level 2) |  |  |  |  |
| Age | 0.01 [-0.00, 0.02] | 0.01 [0.00, 0.02] | 0.01 [-0.00, 0.02] | 0.00 [-0.01, 0.01] |
| Proportion of Solitude | **1.25** [0.09, 2.43] | 0.84 [-0.33, 2.00] | **1.95** [0.65, 3.11] | 1.07 [-0.11, 2.26] |
| Autonomy _Average_ | **0.19** [0.08, 0.30] | 0.08 [-0.03, 0.19] | 0.07 [-0.03, 0.20] | **0.29** [0.18, 0.42] |
| Constraints _Average_ | **-0.13** [-0.24, -0.03] | -0.06 [-0.17, 0.05] | -0.02 [-0.12, 0.10] | -0.08 [-0.19, 0.04] |
|  |  |  |  |  |
| Situational Level (Level 1) |  |  |  |  |
| Autonomy _Situation_ | 0.06 [-0.01, 0.14] | **0.06** [0.00, 0.13] | 0.03 [-0.07, 0.09] | **0.19** [0.10, 0.28] |
| Constraints _Situation_ | **-0.09** [-0.16, -0.01] | 0.01 [-0.05, 0.07] | -0.07 [-0.11, 0.00] | **-0.11** [-0.21, -0.00] |
| ­ |  |  |  |  |
| Cross-Level Interaction |  |  |  |  |
| Age*Autonomy _Situation_ | **-0.01** [-0.01, -0.00] | -0.00 [-0.00, 0.00] | -0.00 [-0.01, 0.00] | **-0.01** (-0.01, -0.00) |
| Age*Constraints _Situation_ | **-0.00** [-0.01, -0.00] | **-0.00** [-0.01, -0.00] | -0.00 [-0.00, 0.00] | -0.01 (-0.01, 0.00) |
|  |  |  |  |  |
| Random Effects | Variance (ΔR²) | | | |
| Intercept | 0.671 (24.2%) | 0.691 (14.2%) | 0.816 (7.3%) | 0.782 (14.9%) |
| Residual | 0.521 (25.2%) | 0.500 (10.3%) | 0.401 (34.5%) | 0.846 (32.0%) |
| Slope _Autonomy Situation_ | 0.007 (76.2%) | 0.000 (91.3%) | 0.002 (2.0%) | 0.010 (51.7%) |
| Slope _Constraints Situation_ | 0.012 (42.9%) | 0.000 (98.5%) | 0.028 (4.1%) | 0.035 (5.1%) |
| *Notes.* Significant parameters (*p* < .05) are printed in bold. *N_Study 1_* = 129. | | | | |

**Supplementary Material S5 – Analyses Including Constraints for Study 2**

Constraints were assessed by asking “What led to the situation and to what extent?” and offering the answering option “constraints of any kind” on a scale ranging from 0 (= *not at all*) to 6 (= *very much*). The mean was 1.86, the standard deviation was 1.37, bivariate correlations are depicted in Supplementary Table S2. A multilevel regression indicated that older adults did not experience less constraints in situations of solitude than younger adults, *γ* = 0.00, *SE* = 0.00, *p* = .839.

| **Supplementary Table S5** | | | | |
| --- | --- | --- | --- | --- |
| *Multilevel Regression Analyses Including the Constraints Construct – Study 1.* | | | | |
| Outcome Variable | Well-being | Self-esteem | Social  Integration | Valence _Situation_ |
| Fixed Effects | Coefficient [95% CI] | | | |
| Intercept | **2.47** [2.40, 2.56] | **4.22** [4.05, 4.38] | **2.19** [2.10, 2.29] | **2.63** [2.51, 2.73] |
|  |  |  |  |  |
| Individual Level (Level 2) |  |  |  |  |
| Age | **0.01** [0.00, 0.01] | **0.02** [0.01, 0.02] | 0.00 [-0.00, 0.01] | -0.01 [-0.01, 0.00] |
| Proportion of Solitude | -0.26 [-0.64, 0.14] | -0.67 [-1.46, 0.15] | -0.19 [-0.70, 0.25] | -0.52 [-1.11, 0.03] |
| Autonomy _Average_ | **0.31** [0.16, 0.41] | **0.39** [0.14, 0.65] | 0.10 [-0.07, 0.24] | **0.47** [0.29, 0.63] |
| Constraints _Average_ | 0.01 [-0.08, 0.13] | 0.17 [-0.04, 0.39] | 0.09 [-0.02, 0.22] | 0.08 [-0.06, 0.23] |
|  |  |  |  |  |
| Situational Level (Level 1) |  |  |  |  |
| Autonomy _Situation_ | **0.24** [0.22, 0.30] | **0.26** [0.19, 0.34] | **0.10** [0.05, 0.16] | **0.20** [0.14, 0.26] |
| Constraints _Situation_ | **-0.04** [-0.07, -0.00] | -0.02 [-0.07, 0.03] | -0.02 [-0.05, 0.02] | **-0.07** [-0.12, -0.04] |
| ­ |  |  |  |  |
| Cross-Level Interaction |  |  |  |  |
| Age*Autonomy _Situation_ | **-0.00** [-0.00, -0.00] | -0.00 [-0.01, 0.00] | **-0.00** [-0.01, -0.00] | **-0.00** [-0.01, -0.00] |
| Age*Constraints _Situation_ | -0.00 [-0.00, 0.00] | 0.00 [-0.00, 0.00] | -0.00 [-0.00, 0.00] | 0.00 [-0.00, 0.00] |
|  |  |  |  |  |
| Random Effects | Variance (ΔR²) | | | |
| Intercept | 0.128 (23.2%) | 0.626 (16.7%) | 0.190 (---) | 0.266 (22.6%) |
| Residual | 0.244 (21.2%) | 0.582 (15.7%) | 0.361 (8.6%) | 0.382 (16.1%) |
| Slope _Autonomy Situation_ | 0.002 (82.4%) | 0.040 (5.3%) | 0.029 (13.0%) | 0.023 (27.5%) |
| Slope _Constraints Situation_ | 0.009 (---) | 0.014 (---) | 0.004 (17.7%) | 0.009 (---) |
| *Notes.* Significant parameters (*p* < .05) are printed in bold. *N_Study 2_* = 115. | | | | |

**Supplementary Material S6 – Analyses Including Constraints for Study 3**

Constraints were assessed by asking “What led to the situation and to what extent?” and offering the answering option “constraints of any kind” on a scale ranging from 0 (= *not at all*) to 6 (= *very much*). Individuals in the *low autonomy* condition reported significantly more constraints than individuals in the *high autonomy* condition, *M(SD)_1_* = 2.73(2.06), *M(SD)_2_* = 1.67(1.67), *t*(324.81) = -4.81, *p* < .001, *d* = -0.53. Bivariate correlations are depicted in Supplementary Table S2.

| **Supplementary Table S6** | | | | | | | | |
| --- | --- | --- | --- | --- | --- | --- | --- | --- |
| *Regression Analyses on the Remembered Experience of Solitude Including the Constraints Construct – Study 3.* | | | | | | | | |
| Outcome Variable | Well-being | | Self-esteem | | Social  Integration | | Valence _Situation_ | |
| Constructs | Coefficient [95% CI] | | | | | | |  |
| Intercept | **3.03** [2.80, 3.26] | **3.60** [3.40, 3.80] | **3.46** [3.25, 3.69] | **3.92** [3.71, 4.13] | **2.67** [2.43, 2.91] | **3.24** [3.02, 3.46] | **2.90** [2.60, 3.19] | **3.64** [3.38, 3.89] |
|  |  |  |  |  |  |  |  |  |
| Condition | **1.29** [0.97, 1.61] | 0.19 [-0.11, 0.49] | **0.98** [0.67, 1.29] | 0.10 [-0.21, 0.42] | **1.18** [0.85, 1.52] | 0.09 [-0.24, 0.41] | **1.81** [1.40, 2.22] | **0.38** [0.00, 0.76] |
| Age | **0.02** [0.00, 0.04] | 0.01 [-0.00, 0.02] | **0.02** [0.00, 0.04] | 0.01 [-0.00, 0.03] | **0.02** [0.01, 0.04] | **0.02** [0.00, 0.03] | 0.01 [-0.01, 0.03] | 0.00 [-0.02, 0.02] |
| Age*Condition | -0.00 [-0.03, 0.01] | -0.00 [-0.02, 0.01] | -0.01 [-0.03, 0.01] | -0.00 [-0.02, 0.01] | -0.01 [-0.03, 0.01] | -0.00 [-0.02, 0.02] | 0.00 [-0.03, 0.03] | 0.01 [-0.01, 0.03] |
|  |  |  |  |  |  |  |  |  |
| Autonomy |  | **0.38** [0.31, 0.45] |  | **0.29** [0.22, 0.36] |  | **0.38** [0.30, 0.45] |  | **0.52** [0.43, 0.60] |
| Constraints |  | **-0.15** [-0.22, -0.08] |  | **-0.15** [-0.22, -0.08] |  | **-0.16** [-0.23, -0.08] |  | **-0.15** [-0.24, -0.06] |
|  |  |  |  |  |  |  |  |  |
| R² | 16.1% | 47.0% | 11.2% | 34.9% | 15.3% | 43.3% | 18.3% | 49.7% |
| *Notes.* Significant parameters (*p* < .05) are printed in bold. The intercept pertains to individuals in the low autonomy condition. *N_Study 3_* = 323. | | | | | | | | |
